# Population-based retrospective cohort study on risk of age-related macular degeneration in people with chronic obstructive pulmonary disease

**DOI:** 10.1038/s41598-021-94657-9

**Published:** 2021-07-23

**Authors:** Pei-Jane Bair, Ning-Yi Hsia, Cheng-Li Lin, Yu-Cih Yang, Te-Chun Shen, Chi‐Yuan Li

**Affiliations:** 1 Graduate Institute of Biomedical Sciences, College of Medicine, China Medical University, No. 91, Hsueh-Shih Road, Taichung, 404 Taiwan; 2 Department of Ophthalmology, Show Chwan Memorial Hospital, No. 542, Section 1, Chung-Shan Road, Changhua, 500 Taiwan; 3 Department of Ophthalmology, China Medical University Hospital, No. 2, Yude Road, Taichung, 404 Taiwan; 4 School of Medicine, College of Medicine, China Medical University, No. 91, Hsueh-Shih Road, Taichung, 404 Taiwan; 5 Management Office for Health Data, China Medical University Hospital, No. 2, Yude Road, Taichung, 404 Taiwan; 6 Division of Pulmonary and Critical Care Medicine, Department of Internal Medicine, China Medical University Hospital, No. 2, Yude Road, Taichung, 404 Taiwan

**Keywords:** Diseases, Health care, Risk factors

## Abstract

Chronic obstructive pulmonary disease (COPD) and age-related macular degeneration (AMD) are both common diseases of the elderly people. COPD induced systemic inflammation and hypoxia may have an impact on the development of AMD. This study investigated the possible association between COPD and subsequent risk of AMD. A retrospective cohort study was conducted based on the data from the National Health Insurance Research Database in Taiwan. The COPD cohort comprised 24,625 adult patients newly diagnosed during 2000–2012, whereas age-, gender-, and the year of diagnosis-matched non-COPD cohort comprised 49,250 individuals. Incident AMD was monitored to the end of 2013. A Cox proportional hazards model was applied to evaluate the risk of AMD. The COPD cohort showed 1.25 times higher AMD incidence than the non-COPD cohort (4.80 versus 3.83 per 1000 person-years, adjusted hazard ratio (HR) = 1.20 [95% confident interval (CI) = 1.10–1.32]). Stratified analyses for age, gender, and presence of comorbidity resulted in significant adjusted HRs in most subgroups. Further analysis revealed that the COPD group had an increased risk of both the exudative and non-exudative types of AMD (adjusted HRs = 1.49 [95% CI = 1.13–1.96] and 1.15 [95% CI = 1.05–1.26], respectively). COPD patients have an increased risk for AMD development. Clinicians should provide adequate care for the ocular health to these patients.

## Introduction

Age-related macular degeneration (AMD) is a chronic disease of the central retina and a leading cause of vision loss in the elderly worldwide. Generally, early AMD was defined as medium-sized drusen without pigmentary abnormalities; late AMD was defined as the presence of either neovascular AMD or geographic atrophy^1^. In people aged 40 years and older, early and late AMD prevalence rates were estimated at 6.8% and 1.5%, respectively^1^. Advanced age is a primary contributing factor for AMD; more than 10% of people older than 80 have late AMD^2^. Reported ocular risk factors for AMD include previous cataract surgery and hyperopic refraction^3–5^. Other potential risk factors linked to AMD are genetics and family history, obesity, lifestyle, diet and nutrition, smoking, and cardiovascular diseases, and their associated factors^6–10^. Therefore, systemic diseases could have an impact on the development of AMD.

Chronic obstructive pulmonary disease (COPD) is characterized by persistent respiratory symptoms and airflow limitation. Pathological changes could occur in the airways, lung parenchyma, and pulmonary vasculature^11^. Chronic inflammation in the respiratory system is a typical feature of COPD; however, it is not limited to the respiratory system and has extensive effects^12^. Systemic inflammation from COPD could play an essential role in multiple comorbid conditions, such as cardiovascular diseases, diabetes, metabolic syndrome, osteoporosis, depression, sarcopenia, and normocytic anemia^13–15^. In addition, COPD could lead to chronic respiratory impairment and hypoxia that contribute to various comorbid conditions^16^.

Only a few studies focused on the relationship between COPD and AMD. Klein et al. conducted a population-based cohort study and reported that a history of emphysema at baseline was associated with a 5.12-fold risk for exudative AMD occurrence^17^. Subsequently, the authors extended the study and reported that a history of emphysema at baseline was associated with a 3.65-fold risk for the 15-year exudative AMD cumulative incidence^18^. However, the same study group conducted another cross-sectional study and failed to identify an association between poor lung function or percent emphysema on computed tomography (CT) scans and AMD presence^19^. In addition, Zlateva et al. conducted a case–control study to investigate comorbid conditions in individuals with and without neovascular AMD^20^. They found that patients with neovascular AMD were significantly associated with COPD and emphysema compared with those without neovascular AMD.

Comorbidities and complications of COPD have been gradually recognized and emphasized as they play an important role in life quality maintenance and disease control. The association between COPD and AMD was not well-established due to the inconsistency of results from previous studies, relatively small samples, less involvement of the Asian population, and most study designs were cross-sectional or case–control. Therefore, this study aimed to conduct a nationwide population-based cohort study using the National Health Insurance Research Database (NHIRD) in Taiwan to further clarify the association of COPD with AMD development.

## Materials and methods

### Data source

The National Health Insurance (NHI) program covers more than 99% of population in Taiwan since 1995. NHIRD is set up and managed by the National Health Research Institutes of Taiwan. For the present study, we used a subset of NHIRD, Longitudinal Health Insurance Database 2000 (LHID 2000), which included detailed medical information of 1,000,000 people randomly selected in 2000. LHID2000 contains all data on demographic characteristics, diagnostic codes, medications, and medical procedures and surgeries. This study obtained approval from the Research Ethics Committee of China Medical University and Hospital (CMUH-104-REC2-115). All methods were performed in accordance with the relevant guidelines and regulations. The reports were in accordance with the Strengthening the Reporting of Observational Studies in Epidemiology (STROBE) guideline. Informed consent was unnecessary for the de-identified data and waived by the Research Ethics Committee of the China Medical University and Hospital.

### Study population

**Figure 1:**
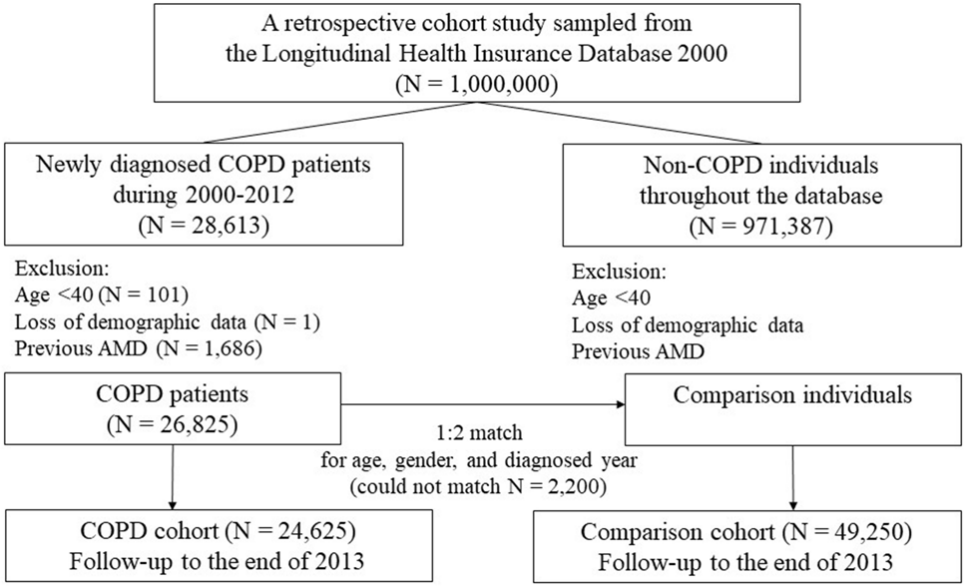
Flowchart of the study design.

The COPD cohort included newly diagnosed patients with COPD (identified from ICD-9-CM code 496) between 2000 and 2012. The index date was defined as the date when the diagnosis was made. We excluded patients with age < 40 years, those with incomplete age or gender information, and those diagnosed with AMD before the index date. The comparison cohort included individuals without COPD that were age-, gender-, and index year-matched with the COPD cohort. The exclusion criteria were the same for the non-COPD cohort as for the COPD cohort (Fig. 1). All participants were monitored until the following events: development of AMD, withdrawal from the NHI system, death, or the end of 2013.

### Study outcome and comorbidities

The primary outcome of interest was the incidence of AMD (identified from ICD-9-CM code 362.50, 362.51, and 362.52). The AMD was further classified into exudative type (identified from ICD-9-CM code 362.52) and non-exudative type (identified from codes 362.50 and 362.51)^21^. In addition, we collected several comorbidities associated to AMD as the potential confounders. These comorbidities included hypertension (identified from ICD-9-CM codes 401–405), diabetes (identified from ICD-9-CM code 250), hyperlipidemia (identified from ICD-9-CM code 272), chronic liver disease and cirrhosis (CLD) (identified from ICD-9-CM code 571), chronic kidney disease (CKD) (identified from ICD-9-CM code 585), and rheumatic diseases (identified from ICD-9-CM codes 446.5, 710.0–710.4, 714.0–714.2, 714.8, and 725). All comorbidities were traced back and recognized from 1995 to the index date (baseline).

### Statistical analysis

A Chi-squared test was used for the inter-group comparison of the distribution of baseline characteristics (categorical variables), whereas a *t*-test was used in cases of continuous data. Variates (COPD, age, gender, and comorbidities) were evaluated for the risk of AMD development. Cox proportional hazard models were performed to determine the adjusted HRs (aHRs) with 95% CI after controlling for variates, significant in the univariate model. Furthermore, stratified analyses for age, gender, and presence of comorbidity were performed to make intergroup comparison. Cox proportional hazard models were also applied here. Based on the Kaplan–Meier curve, both cohorts’ cumulative AMD incidence was analyzed, and a log-rank test was performed to make inter-group comparison. Data analysis was conducted using SAS statistical software (Version 9.4 for Windows; SAS Institute, Inc., Cary, NC, USA). A two-tailed *p* value < 0.05 was considered statistically significant.

## Results

We recruited a COPD cohort consisting of 24,625 patients and a comparison cohort comprising 49,250 individuals (Table 1). Both groups showed similar age and gender distributions. The mean age ± standard division was 67.9 ± 11.7 years in the COPD cohort and 67.0 ± 11.9 years in the comparison cohort. In both cohorts, 67.4% of the individuals were men. Patients with COPD had significantly higher prevalence of hypertension (65.9% vs. 52.3%), hyperlipidemia (34.5% vs. 29.4%), CLD (33.4% vs. 24.9%), diabetes (13.2% vs. 10.3%), rheumatic disease (4.80% vs. 3.23%), and CKD (4.45% vs. 3.11%) than the non-COPD comparison group.

**Table 1:** Baseline characteristics for individuals with and without COPD.

	COPD				*p*-value
	No		Yes		
	N = 49,250		N = 24,625		
	n	%	n	%	
**Age**					0.99
40–64	18,288	37.1	9144	37.1	
65–74	15,786	32.1	7981	32.4	
≥ 75	15,176	30.8	7500	30.5	
Mean ± SD	67.0	± 11.9	67.9	± 11.7	0.001
**Gender**					0.99
Women	16,070	32.6	8035	32.6	
Men	33,180	67.4	16,590	67.4	
**Comorbidity**					
Hypertension	25,737	52.3	16,227	65.9	< 0.001
Diabetes mellitus	5077	10.3	3241	13.2	< 0.001
Hyperlipidemia	14,499	29.4	8495	34.5	< 0.001
CLD	12,244	24.9	8235	33.4	< 0.001
CKD	1533	3.11	1096	4.45	< 0.001
Rheumatic disease	1589	3.23	1181	4.80	< 0.001

CKD, chronic kidney disease; CLD, chronic liver disease and cirrhosis; COPD, chronic obstructive pulmonary disease; SD, standard deviation.

The overall AMD incidence rate was higher in the COPD group than in the non-COPD group (4.80 vs. 3.83 per 1000 person-years) (Table 2). The corresponding aHR in the COPD group compared with the non-COPD group, adjusted for age and comorbidities, was 1.20 (95% CI = 1.10–1.32). When compared with individuals aged 40–64, those aged 65–74 years had a 2.66 higher (95% CI = 2.38–2.97) and those aged ≥ 75 had a 2.71-fold higher risk of AMD (95% CI = 2.32–3.17). Individuals with rheumatic disease (aHR = 1.33, 95% CI = 1.09–1.62), diabetes (aHR = 1.26, 95% CI = 1.11–1.43), CLD (aHR = 1.26, 95% CI = 1.14–1.38), hypertension (aHR = 1.25, 95% CI = 1.13–1.38), and hyperlipidemia (aHR = 1.25, 95% CI = 1.14–1.38) had a higher risk than individuals without these comorbidities.

**Table 2:** Incidences and hazard ratios of age-related macular degeneration for potential risk factors.

	Event	PY	Rate^a^	Crude HR (95% CI)	Adjusted HR^b^ (95% CI)
**COPD**					
No	1360	355,359	3.83	1.00	1.00
Yes	760	158,231	4.80	1.26 (1.15–1.38)***	1.20 (1.10–1.32)***
**Age**					
40–64	432	216,521	2.00	1.00	1.00
65–74	1421	248,636	5.72	2.88 (2.58–3.21)***	2.66 (2.38–2.97)***
≥ 75	267	48,433	5.51	2.83 (2.43–3.30)***	2.71 (2.32–3.17)***
**Gender**					
Women	669	170,903	3.91	1.00	1.00
Men	1451	342,687	4.23	1.08 (0.99–1.19)	
**Comorbidity**					
Hypertension					
No	692	242,382	2.85	1.00	1.00
Yes	1428	271,207	5.27	1.85 (1.69–2.03)***	1.25 (1.13–1.38)***
Diabetes mellitus					
No	1809	465,304	3.89	1.00	1.00
Yes	311	48,285	6.44	1.67 (1.48–1.88)***	1.26 (1.11–1.43)***
Hyperlipidemia					
No	1288	362,817	3.55	1.00	1.00
Yes	832	150,772	5.52	1.56 (1.43–1.70)***	1.25 (1.14–1.38)***
CLD					
No	1407	377,418	3.73	1.00	1.00
Yes	713	136,172	5.24	1.41 (1.21–1.54)***	1.26 (1.14–1.38)***
CKD					
No	2061	501,257	4.11	1.00	1.00
Yes	59	12,333	4.78	1.18 (0.91–1.52)	
Rheumatic disease					
No	2014	496,937	4.05	1.00	1.00
Yes	106	16,652	6.37	1.58 (1.30–1.92)***	1.33 (1.09–1.62)**

CI, confidence interval; CKD, chronic kidney disease; CLD, chronic liver disease and cirrhosis; COPD, chronic obstructive pulmonary disease; HR, hazard ratio; PY, person-years.

**p < 0.01, ***p < 0.001.

^a^Incidence rate per 1000 person-years.

^b^Multivariable analysis including age and comorbidities of hypertension, diabetes, hyperlipidemia, CLD, and rheumatic disease.

Table 3 shows the association between COPD and AMD stratified by age, gender, and comorbidity. The aHRs for AMD in COPD vs. the non-COPD group were 1.20 (95% CI = 0.99–1.46), 1.21 (95% CI = 1.09–1.35), and 1.06 (95% CI = 0.82–1.38) in individuals 40–64, 65–74, and ≥ 75 years, respectively. The aHRs for AMD in the COPD vs. the non-COPD group were 1.02 (95% CI = 0.87–1.21) and 1.29 (95% CI = 1.16–1.44) in women and men, respectively. The aHRs for AMD in the COPD vs. the non-COPD group were 1.67 (95% CI = 1.35–2.07) and 1.13 (95% CI = 1.03–1.25) in individuals without and with any comorbidity, respectively.

**Table 3:** Incidences and hazard ratios of age-related macular degeneration for patients with COPD compared to individuals without COPD.

	COPD						Crude HR (95% CI)	Adjusted HR^b^ (95% CI)
	No			Yes				
	Event	PY	Rate^a^	Event	PY	Rate^a^		
**Age**								
40–64	257	147,376	1.74	175	69,145	2.53	1.46 (1.21–1.77)***	1.20 (0.99–1.46)
65–74	918	173,256	5.30	503	75,379	6.67	1.27 (1.14–1.41)***	1.21 (1.09–1.35)***
≥ 75	185	34,726	5.33	82	13,707	5.98	1.10 (0.85–1.43)	1.06 (0.82–1.38)
**Gender**								
Women	449	117,767	3.81	220	53,135	4.14	1.09 (0.92–1.28)	1.02 (0.87–1.21)
Men	911	237,591	3.83	540	105,096	5.14	1.35 (1.21–1.50)***	1.29 (1.16–1.44)***
**Comorbidity**^**c**^								
No	290	134,125	2.16	122	34,409	3.55	1.65 (1.34–2.04)***	1.67 (1.35–2.07)***
Yes	1070	221,234	4.84	638	123,822	5.15	1.07 (0.97–1.18)	1.13 (1.03–1.25)*

CI, confidence interval; COPD, chronic obstructive pulmonary disease; HR, hazard ratio; PY, person-years.

*p < 0.05, ***p < 0.001.

^a^Incidence rate per 1000 person-years.

^b^Multivariable analysis including age and comorbidities of hypertension, diabetes, hyperlipidemia, CLD, and rheumatic disease.

^c^Individuals with any comorbidity of hypertension, diabetes, hyperlipidemia, CLD, CKD, and rheumatic disease were classified into the comorbidity group.

**Figure 2:**
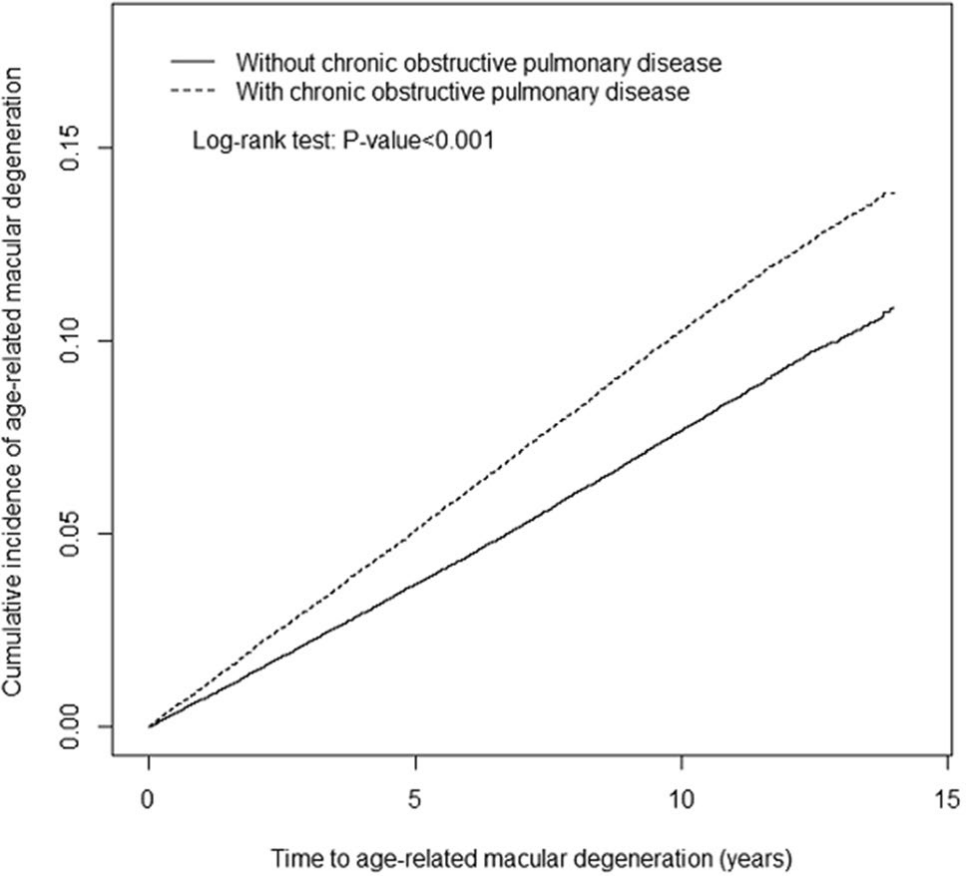
The cumulative incidence of age-related macular degeneration between individuals with and without chronic obstructive pulmonary disease.

We further classified all AMD cases (n = 2120) into exudative type (n = 218) and non-exudative type (n = 1902) (Table 4). The COPD group showed significant risk of both exudative and non-exudative type (aHR = 1.49, 95% CI = 1.13–1.96; aHR = 1.15, 95% CI = 1.05–1.26, respectively) compared with the non-COPD group. The cumulative incidence of AMD in individuals with and without COPD is illustrated in Fig. 2. Compared with the non-COPD group, the log-rank test showed that AMD’s cumulative incidence was significantly higher in the COPD cohort (*p* < 0.001).

**Table 4:** Incidences and hazard ratios of exudative and non-exudative age-related macular degeneration for patients with COPD compared to individuals without COPD.

	COPD	
	No	Yes
**Exudative type**		
Event	129	89
Incidence rate^a^	0.36	0.56
Crude HR (95% CI)	1.00 (reference)	1.56 (1.19–2.04)**
Adjusted HR (95% CI)^b^	1.00 (reference)	1.49 (1.13–1.96)**
**Non-exudative type**		
Event	1231	671
Incidence rate^a^	3.46	4.24
Crude HR (95% CI)	1.00 (reference)	1.23 (1.12–1.35)***
Adjusted HR (95% CI)^b^	1.00 (reference)	1.15 (1.05–1.26)**

CI, confidence interval; COPD, chronic obstructive pulmonary disease; HR, hazard ratio.

**p < 0.01, ***p < 0.001.

^a^Incidence rate per 1000 person-years.

^b^Multivariable analysis including age and comorbidities of hypertension, diabetes, hyperlipidemia, CLD, CKD, and rheumatic disease.

## Discussion

This retrospective population-based cohort study examined the AMD incidence in COPD patients and non-COPD individuals. We showed that COPD patients had a significantly elevated risk of developing AMD than those without COPD. As expected, higher age and the presence of comorbidities increased the AMD risk. Furthermore, hazards of AMD for the COPD cohort compared to non-COPD cohort remained significantly higher in subgroups of 65–74 years, male gender, with any comorbidity, and without comorbidity. Moreover, patients with COPD have a higher risk of developing exudative and non-exudative AMD than those without COPD.

Exact pathogenesis of AMD is unclear but is reportedly caused by a complex multifactorial interaction between genetic, metabolic, functional, and environmental factors. Possible explanations for the association between COPD and AMD are (1) systemic inflammation and (2) hypoxia. AMD can be characterized as a chronic inflammatory state, with local infiltration of inflammatory cells, higher circulatory proinflammatory cytokine levels, and complement components^22^. Inflammatory processes, including innate immune responses, microglial activation, and para-inflammation occurring in the choroid, the retinal pigment epithelium (RPE), and the neuroretina are essential for AMD’s development and progression^23,24^. Hypoxia could induce the failure of energy balance, cellular damage, the release of excitatory neurotransmitters, inflammation, and delayed cell death^25^. Hypoxia can lead to an imbalance between oxidative stress-induced cellular damage and the remodeling process and hypoxia-mediated signaling, such as the hypoxia-inducible factor (HIF-1a) pathway thought to underlie AMD’s development and progression^24–27^.

In previous studies, Klein et al. initially evaluated 3675 persons (Beaver Dam Eye Study, total 15-year follow-up) and found that a history of emphysema at baseline (n = 75), independent of the smoking status, was associated with the incidence of increased retinal pigmentation, RPE depigmentation, and exudative AMD^17,18^. Furthermore, they evaluated another population of 3399 persons (MESA study, cross-sectional) and reported that early AMD was not associated with forced expiratory volume in 1 s (FEV1), FEV1 to forced vital capacity ratio (FEV1/FVC), and percent emphysema on a CT scan^19^. The findings were inconsistent with a former Framingham Eye Study in 1977 that found a higher frequency of AMD in persons with reduced FVC^28^. These studies aimed to check a community population and included very few patients with empyema, whereas our study aimed to establish a large COPD cohort and matched a non-COPD cohort. We could directly compare the differences, with unique and solid results. In another similar study using ICD-9-CM codes from Medicare beneficiaries in the United States, Zlateva et al. evaluated comorbid conditions between a neovascular AMD group and control group (case–control study) and found that patients with neovascular AMD are significantly more likely to have COPD (8.4% vs 6.5%, OR = 1.309, 95% CI = 1.201–1.428) and emphysema (11.6% vs. 9.1%, OR = 1.319, 95% CI = 1.224–1.421)^20^. In our study, we found that patients with COPD are significantly more likely to develop AMD in a 14-year follow-up (5.52% vs. 1.54%). As mentioned above, multiple comorbid conditions in COPD have been recognized and could largely decrease the quality of life of these patients. The findings of this study are a reminder of the importance of ocular health in patients with COPD. Clinicians may pay more attention to ocular health, such as AMD or other ocular disorders, and provide adequate integrated care for the aforementioned patients.

The strengths of the study were to establish nationwide, population-based cohorts (COPD and non-COPD) and follow the incident AMD for near 15 years. The NHIRD in Taiwan is one of the largest nationwide population databases in the world. More important, universal coverage (> 99% of whole population) helps to access all citizens, regardless of socioeconomic background and residential location^29^. Overall, in the present study, we have shown the novel correlation from the real-world practice.

In the present study, the most noticeable potential confounding factor was cigarette smoking. Smoking information was not included in the NHI database; however, the COPD group may include > 50% ex-smokers or current smokers^30,31^. In a recent official report in Taiwan, the current smoking rate of the general population was 19.8% (http://hpa.gov.tw). This represents a possible smoking rate for the non-COPD group. Creating a non-COPD group with a very high smoking rate is difficult, thus we combine COPD and smoking effects to evaluate the risk of AMD development in the study.

Some other limitations should be addressed in the study. First, the ICD-9-CM codes were applied to define COPD, AMD, and comorbidities. An ad hoc committee was set up to prevent errors and violations. In addition, COPD coding in the database has been validated^32,33^. Second, the NHIRD does not include alcohol consumption, dietary pattern, and other environmental factors that may be potential confounding factors. Third, clinical variables such as body weight, pulmonary function parameters, laboratory data (systemic inflammation and hypoxia), image reports, and ocular findings were unavailable. Moreover, the disease severity was not recorded in the database.

In conclusion, patients with COPD may have an increased risk to develop AMD. The underlying mechanisms and the possible causality should be investigated and established. In any case, ocular health may be noticed in patients with COPD.
